# Association of CDKN2A/B Homozygous Deletion and Extent of Resection With Survival of Patients With WHO CNS5 Grade 4 Astrocytoma

**DOI:** 10.1002/acn3.70310

**Published:** 2026-01-15

**Authors:** Jiawei Cai, Guanglin Zhu, Qiu He, Chen Luo, Lingyun Zhuo, Xingfu Wang, Yiming Chen, Xiaoyong Chen, Wendong You, Jiaheng Xu, Yuanxiang Lin, Dezhi Kang, Shuai Wu, Zanyi Wu

**Affiliations:** ^1^ Department of Neurosurgery Neurosurgery Research Institute, the First Affiliated Hospital of Fujian Medical University Fuzhou China; ^2^ Department of Neurosurgery National Regional Medical Center, Binhai Campus of the First Affiliated Hospital, Fujian Medical University Fuzhou China; ^3^ Department of Neurosurgery Huashan Hospital, Shanghai Medical College, Fudan University Shanghai China; ^4^ School of Medical Technology and Engineering Fujian Medical University Fuzhou China

**Keywords:** *CDKN2A/B*, extent of resection, grade 4 astrocytoma, management algorithm, prognosis

## Abstract

**Objectives:**

WHO grade 4 astrocytomas are associated with poor prognosis, and their prognostic factors remain controversial. This study aimed to identify the prognostic factors and develop a management algorithm for these patients.

**Methods:**

This study retrospectively included 151 CNS5 adult grade 4 astrocytomas from two medical centers. The tumors were classified as histologic grade 2/3 astrocytomas with *CDKN2A/B* homozygous deletion (molecular grade 4 astrocytoma, MA4), histologic grade 4 astrocytomas with *CDKN2A/B* homozygous deletion (molecular and histologic grade 4 astrocytoma, MHA4), and histologic grade 4 astrocytomas without *CDKN2A/B* homozygous deletion (histologic grade 4 astrocytoma, HA4). Prognostic factors were identified and incorporated into recursive partitioning analysis (RPA) for survival risk stratification.

**Results:**

Histologic grade 4 astrocytomas with *CDKN2A/B* homozygous deletion, postoperative tumor volume (TV), and chemoradiotherapy were associated with patient survival. RPA identified three groups with distinct prognoses (*p* = 0.001). Group 1 had a median overall survival (OS) of 77.8 months, consisting of MA4 and HA4 with postoperative TV on FLAIR ≤ 28.5 mL. Group 2 had a median OS of 32.2 months, including MA4 and HA4 with postoperative TV on FLAIR > 28.5 mL receiving chemoradiotherapy, or MHA4 with postoperative TV on FLAIR ≤ 28.5 mL. Group 3 had a median OS of 14.9 months, including MA4 and HA4 with postoperative TV on FLAIR > 28.5 mL without chemoradiotherapy, or MHA4 with postoperative TV on FLAIR > 28.5 mL receiving chemoradiotherapy.

**Conclusion:**

Histologic grade 4 astrocytomas with *CDKN2A/B* homozygous deletion confer the worst survival. Maximal or complete resection, as assessed on FLAIR images, is critical to improving outcomes.

## 1 Introduction

*CDKN2A/B*, a gene regulating cell growth, is frequently altered in human cancers and promotes malignant tumor progression [1, 2, 3]. Homozygous deletion of *CDKN2A/B* occurred in 0%–12% of WHO 2 *IDH*‐mutant astrocytoma, 6%–20% of WHO 3 *IDH*‐mutant astrocytoma, and 16%–34% of WHO 4 *IDH*‐mutant astrocytoma [4, 5, 6]. Clinical studies indicated that patients with histologic grade 2/3 *IDH*‐mutant astrocytoma harboring *CDKN2A/B* homozygous deletion exhibit poorer prognoses [7, 8, 9]. The fifth edition of the WHO Classification of Tumors of the Central Nervous System (WHO CNS5) introduced a new category of grade 4 astrocytomas, which includes all *IDH*‐mutant astrocytomas with homozygous CDKN2A/B deletion, regardless of their histopathological features [10, 11]. Accordingly, the current classification of WHO grade 4 astrocytoma actually consists of histologic grade 2/3 astrocytomas with *CDKN2A/B* homozygous deletion (molecular grade 4 astrocytoma, MA4), histologic grade 4 astrocytoma with *CDKN2A/B* homozygous deletion (molecular and histologic grade 4 astrocytoma, MHA4), and histologic grade 4 astrocytomas without *CDKN2A/B* homozygous deletion (histologic grade 4 astrocytoma, HA4). This framework underscores the diagnostic significance of *CDKN2A/B* homozygous deletion in WHO grade 4 astrocytoma.

Although *CDKN2A/B* homozygous deletion is a recognized prognostic factor in histologic grade 2/3 astrocytoma, its role in histologic grade 4 astrocytoma remains controversial [12, 13]. Furthermore, the prognostic determinants of WHO grade 4 astrocytoma are not fully established, complicating the diagnosis and clinical management. Previous studies have demonstrated that maximal resection of lesions, as assessed on T2‐fluid attenuated inversion recovery (T2‐FLAIR) images, improves overall survival (OS) in patients with WHO grade 2/3 astrocytomas [14, 15, 16, 17, 18]. Furthermore, extended resection of IDH‐mutant gliomas enhances both progression‐free survival (PFS) and OS [19, 20]. However, the effect of extent of resection (EOR) on the prognosis of WHO grade 4 astrocytomas has been inadequately explored.

In this study, we retrospectively evaluated the impact of CDKN2A/B homozygous deletion and EOR on the outcomes of patients with WHO grade 4 astrocytomas, aiming to develop a management algorithm through population‐based risk stratification.

## 2 Materials and Methods

### 2.1 Data Sources and Ethics Statement

Clinical data were retrospectively collected from patients with *IDH*‐mutant WHO grade 4 astrocytoma and documented *CDKN2A/B* homozygous deletion status at the First Affiliated Hospital of Fujian Medical University (June 2014 to June 2023) and Huashan Hospital of Fudan University (January 2010 to June 2023). This study protocol was also approved by the Ethics Committee of First Affiliated Hospital of Fujian Medical University (Approval No. MRCTA, ECFAH of FMU[2023]457).

**FIGURE 1:**
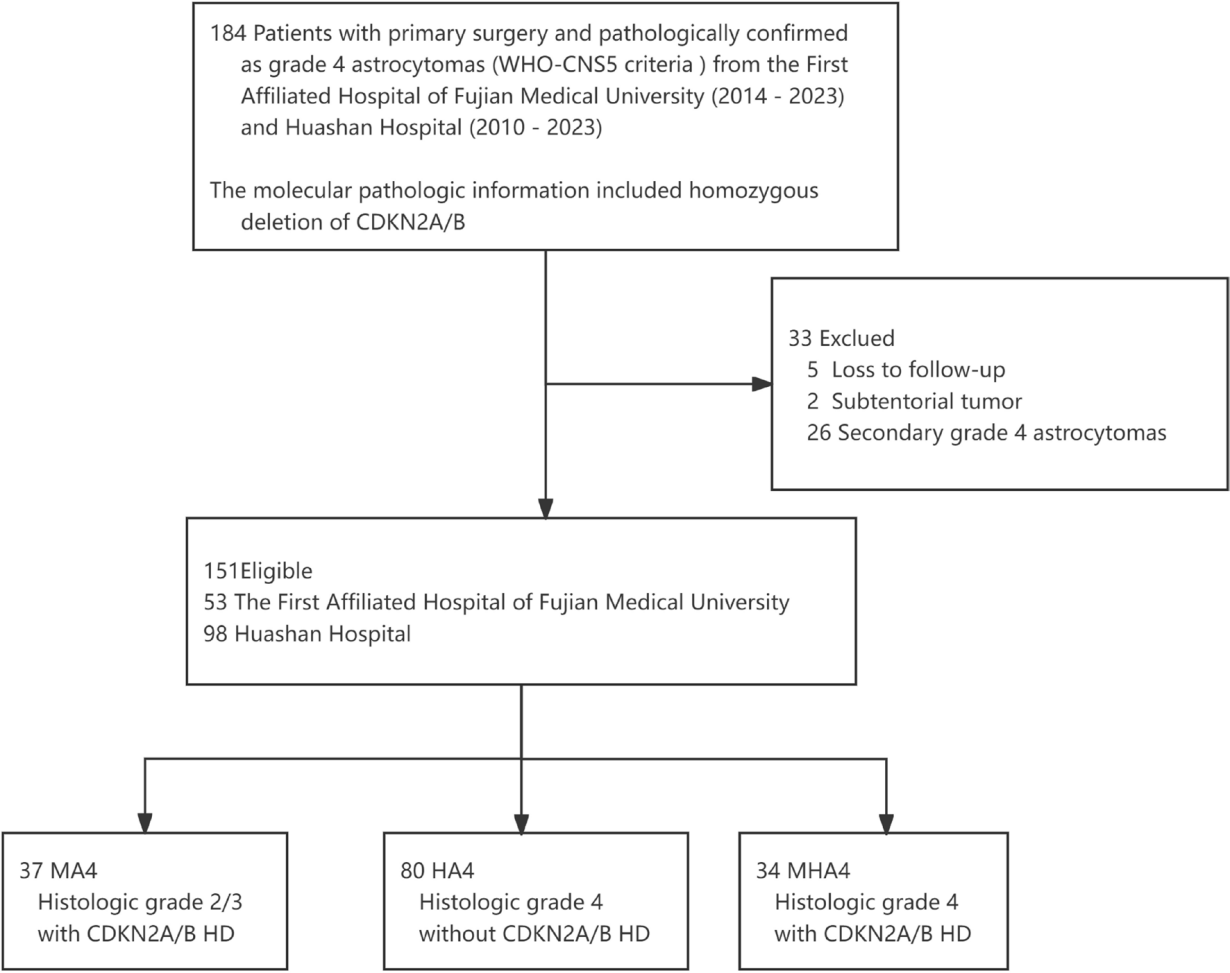
Flow diagram of data collection. HA4, histologic grade 4 astrocytoma; HD, homozygous deletion; MA4, molecular grade 4 astrocytoma; MHA4, molecular and histologic grade 4 astrocytoma.

A total of 184 patients met the initial inclusion criteria (Figure 1). Of these, 33 patients were excluded: 5 due to loss to follow‐up, 2 with subtentorial tumors, and 26 due to multiple operations and incomplete pathological information from initial surgeries. Consequently, 151 patients with *IDH*‐mutant WHO grade 4 astrocytoma were included.

### 2.2 Pathologic Diagnosis and Molecular Analysis

The diagnostic criteria for *IDH*‐mutant WHO grade 4 astrocytoma include diffuse, infiltrative astrocytic gliomas harboring *IDH* mutations, the presence of microvascular hyperplasia or necrosis, or homozygous *CDKN2A/B* deletion, with retained 1p/19q and wild‐type TERT promoter (pTERT) status [10]. Retrospective supplementary tests were conducted on specific specimens, particularly those collected prior to 2021, with informed consent obtained from all patients. *CDKN2A/B* deletion status was detected via fluorescence in situ hybridization (FISH; Anbiping, China) or next‐generation sequencing (NGS). A FISH test was considered positive if more than 30% of cells exhibited *CDKN2A/B* deletion. A copy number variation (CNV) value below 0.6 in NGS analysis was defined as indicative of *CDKN2A/B* deletion.

### 2.3 Analysis of Tumor Volume

Preoperative and postoperative tumor volume (TV) were reconstructed and independently evaluated by a neuroradiologist and a neurosurgeon using Brainlab software (Brainlab, Munich, Germany). TV was assessed on contrast‐enhanced (CE) T1‐weighted images and T2‐FLAIR images, respectively. For patients exhibiting postoperative ischemic regions, combined magnetic resonance imaging (MRI), diffusion‐weighted imaging (DWI), and apparent diffusion coefficient (ADC) sequences were utilized for comprehensive assessment. The EOR was calculated as follows: (preoperative TV − postoperative TV)/preoperative TV × 100%. Intraoperative MRI was the preferred method for assessing postoperative tumor volume and was performed in two scenarios: (1) during resection to assess the extent of tumor removal while resection was still ongoing, and (2) immediately after completion of the surgical procedure to evaluate the extent of tumor resection. Only the post‐resection intraoperative scans were utilized for the analysis of EOR. If intraoperative MRI was not available, postoperative tumor volume was assessed using MRI scans acquired 1 week to 1–2 months after surgery.

### 2.4 Postoperative Chemoradiotherapy

For patients who received radiotherapy, treatment was typically delivered at a total dose of 54–60 Gy in multiple fractions, with a daily fractional dose of 1.8–2.0 Gy, administered 5 days per week over a total duration of 6 weeks. Patients who tolerated radiotherapy received concurrent and adjuvant temozolomide (TMZ) according to the Stupp protocol, as the standard chemotherapeutic regimen [21, 22]. Individualized treatment adjustments were made based on patient tolerance and clinical condition.

### 2.5 Criteria for Tumor Recurrence and Follow‐Up

Tumor recurrence was evaluated according to the criteria for gliomas from the Response Assessment for Neuro‐Oncology (RANO) working group [23]. All patients were monitored through a combination of telephone follow‐up and review of outpatient medical records. PFS was defined as the interval from the date of surgery to the date of radiologically confirmed tumor recurrence, whereas overall survival (OS) was calculated from the date of surgery to the date of death from any cause. For patients who were lost to follow‐up, survival data were censored at the date of the last available contact.

### 2.6 Statistical Analysis

Statistical analyses were performed using SPSS software (version 25.0, IBM Corp. USA) and R software (version 4.2.1, Institute for Statistics and Mathematics, Austria). Continuous variables were analyzed using Student's *t*‐test or the Mann–Whitney test, as appropriate. Categorical variables were analyzed using the chi‐square (*χ* ^2^) test or Fisher's exact test when expected cell counts were small. The Cox proportional hazards model was applied to assess the prognostic significance of variables, calculating hazard ratios (HR) and 95% confidence intervals (CI). Variables with *p* < 0.2 in univariate COX regression were included in multivariate COX regression. Smooth HR analysis determined the cutoffs and benefit breakpoints for TV on FLAIR and EOR. Recursive partitioning analysis (RPA) was used to stratify patients into different survival risk groups. Differences between survival curves were evaluated using the log‐rank test. All statistical tests were two‐sided, with *p* < 0.05 considered statistically significant.

## 3 Results

### 3.1 Patient Characteristics

The clinical characteristics of the 151 patients with WHO grade 4 astrocytoma are summarized in Table 1 and Table S1. The median follow‐up was 31.3 months (95% CI, 18.1–44.4). Tumor recurrence occurred in 90 patients (59.6%), and 73 deaths (48.3%) were recorded during follow‐up. The preoperative CE tumor volume (*p* < 0.001) and postoperative TV on FLAIR (*p* = 0.03) were significantly larger in MHA4 and HA4 compared to MA4.

**TABLE 1:** Clinical characteristics.

Characteristics	MA4 (*n* = 37)	HA4 (*n* = 80)	MHA4 (*n* = 34)	Total (*n* = 151)	*p*
Age (years, mean ± SD)	39.8 ± 10.8	42.0 ± 11.8	45.7 ± 13.1	42.3 ± 13.1	0.110
Gender					0.543
Females (%)	15 (40.5)	41 (51.3)	15 (44.1)	71 (47.0)	
Male (%)	22 (59.5)	39 (48.7)	19 (55.9)	80 (53.0)	
Preoperative KPS					0.124
≤ 70 (%)	0 (0.0)	6 (7.5)	4 (11.8)	10 (6.6)	
80–100 (%)	37 (100.0)	74 (92.5)	30 (88.2)	141 (93.4)	
Tumor emisphere					0.453
Left (%)	20 (54.1)	44 (55.0)	18 (52.9)	82 (54.3)	
Right (%)	16 (43.2)	27 (33.8)	11 (32.4)	54 (35.8)	
Midline/bilateral (%)	1 (2.7)	9 (11.2)	5 (14.7)	15 (9.9)	
Histologic grade					
2 (%)	18 (48.6)	0 (0.0)	0 (0.0)	18 (11.9)	
3 (%)	19 (51.4)	0 (0.0)	0 (0.0)	19 (12.6)	
4 (%)	0 (0.0)	80 (100.0)	34 (100.0)	114 (75.5)	
*CDKN2A/B* Status					
HD (%)	37 (100.0)	0 (0.0)	34 (100.0)	71 (47.0)	
Non‐HD (%)	0 (0.0)	80 (100.0)	0 (0.0)	80 (53.0)	
Postoperative adjuvant therapy					0.986
Non‐RT + CT	7 (18.9)	14 (17.5)	6 (17.6)	27 (17.9)	
RT + CT	30 (81.1)	65 (81.2)	28 (82.4)	124 (82.1)	
Uncharted	0 (0.0)	1 (1.3)	0 (0.0)	0 (0.0)	
Preoperative CE tumor volume (mL, mean ± SD)	5.9 ± 12.9	32.5 ± 28.8	41.6 ± 32.6	28.3 ± 29.9	< 0.001
Preoperative CE tumor volume (mL, median, Q1; Q3)	0 (0–4)	23.0 (7–57)	18.0 (37–70)	20.0 (2–49)	
Preoperative TV on FLAIR (mL, mean ± SD)	111.4 ± 57.7	129.5 ± 62.6	135.9 ± 47.1	126.3 ± 58.6	0.213
Preoperative TV on FLAIR (median, Q1; Q3)	106.0 (75.5–151.0)	132.0 (82.8–167.8)	143.5 (90.8–171.3)	129.0 (87.5–166.0)	
Postoperative CE Tumor volume (mL, mean ± SD)	0.7 ± 3.5	1.2 ± 7.3	2.3 ± 4.5	1.3 ± 6.0	< 0.001
Postoperative CE Tumor volume (mL, median, Q1; Q3)	0 (0–0)	0 (0–0)	0 (0–3.0)	0 (0–0)	
Postoperative TV on FLAIR (mL, mean ± SD)	27.8 ± 32.7	43.5 ± 47.4	44.3 ± 27.1	39.9 ± 40.6	0.030
Postoperative TV on FLAIR (mL, median, Q1; Q3)	20.5 (6.0–36.0)	28.0 (7.0–60.0)	45.0 (22.3–62.0)	28.0 (9.5–54.5)	
Extent of CE Tumor Resection (%, median, Q1; Q3)	100 (100–100)	100 (100–100)	100 (92.0–100)	100 (100–100)	0.003
Extent of Tumor Resection on FLAIR (%, median, Q1; Q3)	82.0 (64.0–94.3)	79.0 (62.0–90.0)	67.0 (36–83)	78.0 (58.8–90.2)	0.044

Abbreviations: CE, contrast‐enhanced; CT, chemotherapy; FLAIR, fluid‐attenuated inversion recovery; HD, homozygous deletion; KPS, Karnofsky performance scale; RT, radiotherapy; SD, standard deviation; TV, tumor volume.

Among the 127 patients with available data on EOR of the CE tumor, 107 (84.3%) achieved total resection. The total CE tumor resection rates were 28/30 (93.3%) in MA4, 61/69 (88.4%) in HA4, and 18/28 (64.3%) in MHA4. Both MA4 and HA4 exhibited significantly higher total CE tumor resection rates compared to MHA4 (*p* = 0.003). Similarly, the extent of tumor resection on FLAIR was greater in MA4 and HA4 than in MHA4 (*p* = 0.044).

### 3.2 Survival Outcome and Prognostic Factors

Multivariate Cox regression analysis identified MHA4 and postoperative TV on FLAIR as independent risk factors for both recurrence and mortality. In contrast, chemoradiotherapy significantly reduced the risk of recurrence (HR = 0.336; 95% CI, 0.173–0.654, *p* = 0.001) and death (HR = 0.208; 95% CI, 0.101–0.428, *p* < 0.001), as presented in Table 2. The EOR on FLAIR was also associated with prognosis; however, due to its strong covariance with postoperative TV on FLAIR (correlation coefficient −0.83, *p* < 0.001), only postoperative TV on FLAIR was retained in the final COX regression model.

**TABLE 2:** COX regression analysis of PFS and OS in 151 patients.

Characteristics	HR	PFS	*p*	HR	OS	*p*
		95% CI			95% CI	
Age	1.013	0.990–1.036	0.267	1.015	0.991–1.041	0.226
Gender (vs. female)	0.601	0.350–1.032	0.065			
Preoperative KPS score	0.983	0.955–1.012	0.254	0.996	0.959–1.033	0.819
Tumor hemisphere						
Left	1.000	—		1.000	—	
Right	1.193	0.658–2.163	0.561	1.417	0.719–2.791	0.314
Midline/bilateral	1.552	0.651–3.698	0.321	0.931	0.363–2.388	0.882
Group						
MA4	1.000	—		1.000	—	
HA4	1.300	0.627–2.696	0.480	0.948	0.415–2.165	0.353
MHA4	3.433	1.37–8.601	0.008	5.732	2.205–14.902	< 0.001
Postoperative adjuvant therapy						
Non‐RT + CT	1.000	—		1.000	—	
RT + CT	0.336	0.173–0.654	0.001	0.208	0.101–0.428	< 0.001
Preoperative CE tumor volume	1.003	0.993–1.013	1.003	1.002	0.992–1.015	0.568
Postoperative TV on FLAIR	1.008	1.001–1.015	0.020	1.015	1.007–1.022	< 0.001

Abbreviations: CE, contrast‐enhanced; CT, chemotherapy; FLAIR, fluid‐attenuated inversion recovery; HR, hazard ratio; KPS, Karnofsky performance scale; PFS, progression‐free survival; RT, radiotherapy; TV, tumor volume.

**FIGURE 2:**
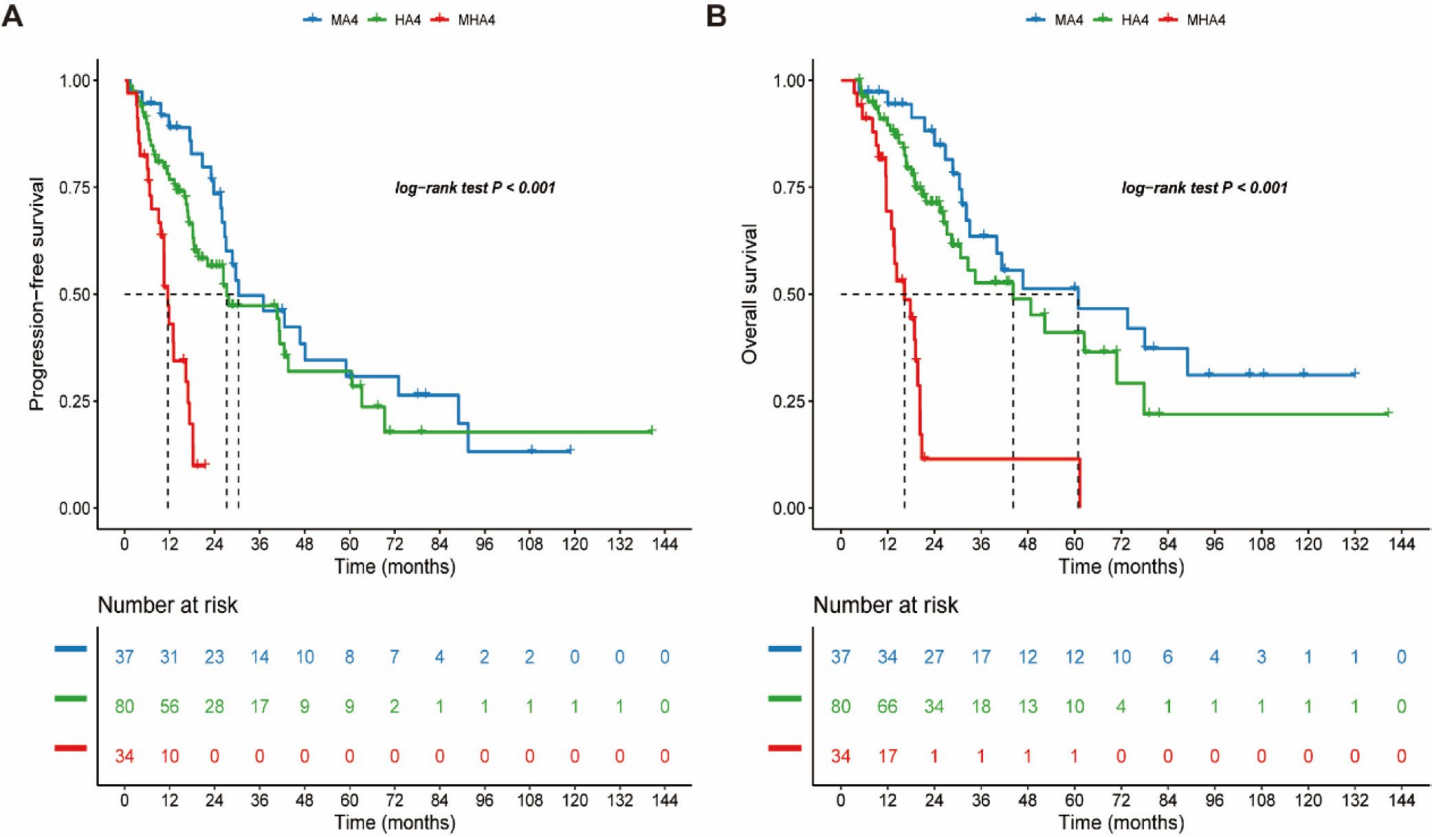
Kaplan–Meier (KM) curves of WHO Grade 4 astrocytomas based on homozygous deletion status of *CDKN2A/B* and histologic grade. (A) KM curves (PFS) in the three groups. (B) KM curves (OS) in the three groups. HA4, Histologic grade 4 without *CDKN2A/B* homozygous deletion; MA4, Histologic grade 2/3 with *CDKN2A/B* homozygous deletion; MHA4, Histologic grade 4 with *CDKN2A/B* homozygous deletion.

Kaplan–Meier (KM) survival curve (Figure 2) demonstrated that the median PFS was 30.4 months (95% CI, 13.3–47.4) for MA4 and 27.2 months (95% CI, 8.0–46.4) for HA4, with no significant difference between the two groups (*p* = 0.305). In contrast, MHA4 exhibited a significantly shorter median PFS of 11.5 months (95% CI, 9.6–13.5; *p* < 0.001). Similarly, the median OS for MHA4 was 16.3 months (95% CI, 10.2–22.4), markedly shorter than that of MA4 (60.0 months; 95% CI, 30.9–89.1) and HA4 (44.2 months; 95% CI, 23.7–67.4; *p* < 0.001). No significant difference in OS was observed between MA4 and HA4 (*p* = 0.208).

Collectively, these findings indicate that even among WHO grade 4 astrocytomas sharing the same *CDKN2A/B* deletion status, histologic grade remains a key determinant of prognosis. The marked survival difference between HA4 and MHA4 further suggests that *CDKN2A/B* homozygous deletion may stratify prognostic risk within histologic grade 4 astrocytomas.

### 3.3 Effect of Extent of Resection on the Prognosis of WHO Grade 4 Astrocytoma

**FIGURE 3:**
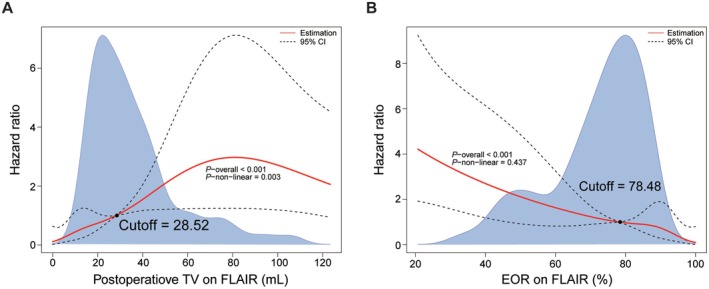
Flexible risk ratio curve analysis of postoperative FLAIR on patients with WHO Grade 4 Astrocytoma. (A) The cutoff point of postoperative TV on FLAIR for OS. (B) The cutoff point of EOR on FALIR for OS. EOR, extent of resection; FLAIR, fluid‐attenuated inversion recovery; OS, overall survival; TV, tumor volume.

A flexible risk ratio curve (SmoothHR) analysis (Figure 3) revealed that postoperative TV on FLAIR significantly impacted OS in a nonlinear manner (*p* < 0.001) (Figure 3A). Patients with a postoperative TV on FLAIR less than 28.5 mL (HR = 1.0) exhibited a significant survival advantage. Among the 132 patients with available postoperative TV on FLAIR data, 67 (50.8%) achieved this cutoff, including 21/32 (65.6%) in the MA4 group, 37/70 (52.9%) in the HA4 group, and 9/30 (30.0%) in the MHA4 group. Additionally, the EOR on FLAIR demonstrated a linear relationship with survival (*p* < 0.001). Increasing EOR correlated with reduced mortality risk, with resections exceeding 78.48% providing significant survival benefits (Figure 3B). Consistent results were observed for progression‐free survival (PFS), with cutoff thresholds of postoperative TV on FLAIR < 28.5 mL and EOR on FLAIR > 78.48%, both closely matching those identified for OS. These findings underscore the prognostic importance of achieving maximal resection in WHO grade 4 astrocytomas.

### 3.4 Risk Stratification for WHO Grade 4 Astrocytoma

**FIGURE 4:**
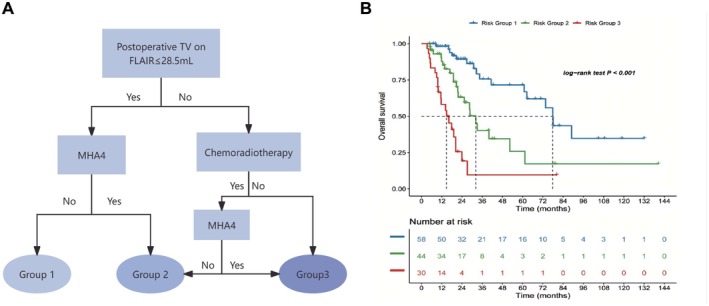
Risk stratification of WHO Grade 4 astrocytoma and overall survival of three risk groups. (A) Three risk groups were identified by recursive partitioning analysis for WHO grade 4 astrocytoma based on FLAIR, CDKN2A/B homozygous deletion and histologic grade combination grouping, and chemoradiotherapy. Group 1 (best median OS 77.8 months) included patients in MA4 or HA4 with postoperative TV on FLAIR ≤ 28.5 mL. Group 2 (intermediate median OS 32.2 months) consisted of patients in MA4 or HA4 who received chemoradiotherapy with postoperative TV on FLAIR > 28.5 mL and patients in MHA4 with postoperative TV on FLAIR ≤ 28.5 mL. Group 3 (worst median OS 14.9 months) included patients with postoperative TV on FLAIR > 28.5 mL who did not receive chemoradiotherapy and patients in MHA4 with postoperative TV on FLAIR > 28.5 mL and chemoradiotherapy. (B) Kaplan–Meier curves for OS by the three risk groups.

RPA was performed on 132 patients with available postoperative TV on FLAIR, using a dichotomized postoperative TV threshold of 28.5 mL together with clinical variables including receipt of chemoradiotherapy and histomolecular subgroup (MA4, HA4, MHA4). The RPA yielded three distinct prognostic groups (Figure 4A): Group 1 (58 patients, median OS 77.8 months) included MA4 or HA4 patients with postoperative TV on FLAIR ≤ 28.5 mL. Group 2 (44 patients, median OS 32.2 months) consisted of MA4 or HA4 patients who received chemoradiotherapy but had postoperative TV on FLAIR > 28.5 mL, and MHA4 patients with postoperative TV on FLAIR ≤ 28.5 mL. Group 3 (30 patients, median OS 14.9 months) included patients with postoperative TV on FLAIR > 28.5 mL who did not receive chemoradiotherapy, and MHA4 patients with postoperative TV on FLAIR > 28.5 mL despite receiving chemoradiotherapy. Notably, MHA4 patients with postoperative TV on FLAIR ≤ 28.5 mL did not attain the superior prognosis observed in Group 1, suggesting that histologic grade (presence of histologic grade 4 features in the setting of CDKN2A/B homozygous deletion) may attenuate the prognostic benefit conferred by aggressive resection that is evident in MA4 and HA4. KM analysis of the three RPA‐defined groups (Figure 4B) indicated distinct survival outcomes: median OS was 77.8 months (95% CI, 50.4–105.3) for Group 1, 32.2 months (95% CI, 26.6–37.8) for Group 2, and 14.9 months (95% CI, 8.1–21.6) for Group 3 (*p* = 0.001).

## 4 Discussion

The WHO CNS5 has refined the definition of grade 4 astrocytoma, improving our understanding of its underlying biological characteristics and clinical behavior. Nevertheless, clinicians face challenges in treatment due to a lack of robust evidence‐based guidelines [6, 24]. In this study, we demonstrated that *CDKN2A/B* homozygous deletion exerts a profound adverse effect on the prognosis of WHO grade 4 astrocytoma, even within histologically similar populations. Moreover, we identified the clinically actionable thresholds for the EOR associated with significant survival benefits—namely, a postoperative TV on FLAIR ≤ 28.5 mL and an EOR on FLAIR > 78.48%. These quantitative parameters provide potential benchmarks for surgical planning and postoperative assessment. Furthermore, RPA allowed us to integrate molecular subtype, postoperative tumor burden, and adjuvant therapy into a unified risk‐stratification framework. This model delineated three prognostic groups with distinct survival outcomes, offering a preliminary algorithm for individualized management of WHO grade 4 astrocytoma. Collectively, these findings highlight the complementary prognostic value of molecular alterations and volumetric surgical metrics in guiding treatment decisions and clinical trial design.

Compared to histologic grade 2/3 astrocytoma, research on *CDKN2A/B* in histologic grade 4 astrocytoma has been limited and yielded inconsistent results. Satomi et al. found that *CDKN2A* deletion was negatively associated with OS in *IDH*‐mutant grade 3 astrocytoma (*n* = 4/35), but not in *IDH*‐mutant grade 4 gliomas (*n* = 13/27) [25]. Conversely, Marker et al. suggested that *CDKN2A/B* is a survival predictor in grade 4 astrocytoma, but not in grade 2/3 [12]. This inconsistency may arise from the use of heterogeneous detection methods with varying sensitivity and specificity across studies [26, 27, 28, 29]. Furthermore, the inclusion of secondary *IDH*‐mutant grade 4 astrocytoma, which are known to have shorter survival and limited therapeutic options, may have further confounded the results [30]. The relatively low incidence of grade 4 astrocytoma also constrains sample size and statistical power in most reports [6, 31, 32].

In our cohort of 117 patients with grade 4 astrocytoma, *CDKN2A/B* homozygous deletion was significantly associated with worse outcomes, with median PFS and OS of 11.5 and 16.3 months, respectively, compared with 27.2 and 44.2 months in those without deletion (both *p* < 0.001). This findings reinforce the unfavorable prognostic impact of *CDKN2A/B* homozygous deletion and provide additional evidence supporting its role as a molecular determinant of survival within histologically uniform WHO grade 4 astrocytomas.

Surgical resection remains the cornerstone of gliomas management, and the therapeutic benefit of resection varies across distinct molecular subtypes [33, 34, 35]. Consequently, individualized surgical strategies based on molecular features are increasingly recognized as a future direction for glioma treatment. In the present study, we identified a quantitative threshold of resection associated with improved survival in WHO grade 4 astrocytoma. The FLAIR sequence was utilized for assessing the EOR for several reasons. First, some MA4 patients did not exhibit CE lesions, making FLAIR‐based volumetry more representative of the tumor burden. Second, WHO grade 4 astrocytoma typically achieve a higher rate of total CE tumor resection, with 84.3% of patients in our cohort achieving total CE tumor resection. Although FLAIR hyperintensity may encompass regions of peritumoral edema, current imaging techniques still face challenges in delineating the true tumor boundaries. Despite this, FLAIR provides a reproducible and routinely available imaging sequence, making it a pragmatic surrogate for assessing residual tumor volume and surgical radicality.

When analyzing tumors with smaller residual volumes, we identified an EOR on FLAIR exceeding 78.48% as the threshold associated with significant survival benefit. Consistent with our findings, Vivas et al. reported in glioblastoma that resection of more than 20% of the non–contrast‐enhancing (NCE) tumor improved survival, whereas no further benefit was observed beyond 60% resection of the NCE component [36] In our cohort, flexible risk ratio curve analysis indicated a linear correlation between the EOR and prognosis for *IDH*‐mutant grade 4 astrocytoma. Survival benefits increased progressively with greater EOR, surpassing the 78.48% threshold and continuing up to complete tumor resection. These results underscore the critical role of maximal safe surgical resection in optimizing outcomes for the patients with grade 4 astrocytoma.

In our study, WHO grade 4 astrocytomas were stratified into three distinct risk groups. MA4 or HA4 patients with postoperative TV on FLAIR ≤ 28.5 mL exhibited the longest OS, whereas MHA4 patients with postoperative TV on FLAIR > 28.5 mL, regardless of receiving chemoradiotherapy, had the shortest OS. This risk stratification provides valuable guidance for clinical prognosis prediction and may inform individualized treatment strategies for WHO grade 4 astrocytoma patients. Notably, MHA4 patients with postoperative TV on FLAIR ≤ 28.5 mL were classified into Group 2, representing an intermediate prognosis, rather than Group 1 with the most favorable prognosis. This observation indicates that the impact of surgical resection differs across molecular subtypes and emphasizes the critical importance of achieving maximal safe resection in WHO grade 4 astrocytoma.

However, this study has several limitations. Only supratentorial astrocytomas were included, as infratentorial tumors exhibit distinct molecular phenotypes and prognoses [37] While the RPA results suggested potential differences in optimal surgical resection strategies among subgroups, the retrospective, single‐center design and limited sample size precluded further exploration of subgroup‐specific resection thresholds and may have introduced selection or analytical bias. Although the cutoff values of postoperative FLAIR volume and EOR were statistically determined, such decimal‐specific thresholds may not reflect real‐world clinical precision. Clinically, approximate ranges may be more practical and generalizable. Additionally, although data were collected from two centers, an external validation cohort was not available due to the low incidence of *CDKN2A/B* homozygous deletion in astrocytomas. These factors, along with the heterogeneity among MA4, HA4, and MHA4 subgroups, may limit the generalizability of our findings.

In conclusion, histologic grade 4 astrocytomas with *CDKN2A/B* homozygous deletion are associated with the poorest survival among WHO grade 4 astrocytomas. Maximal or complete resection of the lesion on FLAIR should be prioritized to achieve a favorable prognosis.

## Author Contributions

Study design: J.C., D.K., S.W., Z.W. Data acquisition: J.C., G.Z., Q.H., C.L., X.C., W.Y., J.X. Methodology: J.C., X.W. Statistical analysis: L.Z., Y.C. Data interpretation: L.Z., Y.L., Z.W. Manuscript preparation: J.C., G.Z., Q.H. Manuscript editing: D.K., S.W.

## Funding

This work was supported by grants from Natural Science Foundation of Fujian Province (No. 2023J01602), Joint Funds for the Innovation of Science and Technology, Fujian Province (No. 2025Y9194), and Neurosurgery Department High‐Level Innovation Construction Project for Major Research at the Neurological Diseases Center (No. 2023YSJYX‐ZD‐2).

## Conflicts of Interest

The authors declare no conflicts of interest.

## Supporting information


**Table S1:** Supplementary clinical characteristics.

## Data Availability

The data that support the findings of this study are available from the corresponding author upon reasonable request.
